# Genome-Wide Analysis and Expression Profiling of Trehalose-6-Phosphate Phosphatase (TPP) in *Punica granatum* in Response to Abscisic-Acid-Mediated Drought Stress

**DOI:** 10.3390/plants12173076

**Published:** 2023-08-28

**Authors:** Fatima Omari Alzahrani

**Affiliations:** Department of Biology, Faculty of Sciences, Al-Baha University, Al-Baha 65729, Saudi Arabia; drfatimaomari@gmail.com or fsalomari@bu.edu.sa

**Keywords:** trehalose, TPP, gene family, genome-wide study, expression analysis

## Abstract

Trehalose, a nonreducing disaccharide, has been linked to plant growth and development as well as stress response. The enzyme trehalose-6-phosphate phosphatase (TPP) plays a crucial role in the production of trehalose in higher plants. This study identified a total of seven TPP family genes within the pomegranate species (*PgTPP1*–*PgTPP7*). Three subgroups of the seven PgTPPs were identified through phylogenetic analysis. The gene length, coding sequence (CD) length, and chromosomal location of the *PgTPP* genes were studied. In addition, the PgTPP proteins’ length, isoelectric point (Ip), grand average of hydropathicity (GRAVY), conserved domains, conserved motifs, synteny, and phylogenetic relationships with Arabidopsis and tomato TPP proteins were examined. The cis-acting elements in the promoter region and the expression of the *PgTPP* genes under abscisic acid (ABA)-mediated drought stress as well as the differences in expression in the root, flower, and leaf tissues were also assessed. The *PgTPP2* and *PgTPP5* genes are involved in the response to abscisic-acid-mediated drought stress, as shown by drought-mediated stress transcriptomes. The *PgTPP1* and *PgTPP2* genes were expressed only in floral tissue and roots, respectively. The remaining *PgTPP*s did not exhibit any significant alterations in gene expression in roots, flowers, or leaves. The current study has the potential to provide a comprehensive understanding of the biological characteristics of PgTPP proteins in various developmental processes and their role in the pomegranate plant’s response to different stressors. However, further research is required to explore their precise biological role. Hence, conducting a comprehensive functional validation study on PgTPPs could contribute to the development of stress-resistant agricultural cultivars.

## 1. Introduction

Enhancing agricultural productivity in diverse environmental conditions, including favorable and unfavorable field conditions, is imperative for enhancing global food security. Nevertheless, it is challenging to simultaneously enhance agricultural output potential and resilience due to the inherent conflict between the factors responsible for stress tolerance and productivity [1].

Plants have acquired the ability to tolerate environmental stress through complex molecular mechanisms that enable them to respond effectively to such stressors. Diverse regulatory networks have the potential to enhance the accumulation of osmotic substances, induce stomatal closure, and restrict the process of photosynthesis [2].

Trehalose, a non-reducing disaccharide, is present in various organisms such as algae, bacteria, plants, fungi, and invertebrates, where it functions as a carbon source, structural component, and stress resistance agent [3].

The significance of trehalose metabolism lies in the fact that it occurs across multiple species and its involvement in various biosynthetic pathways. The metabolic pathway that is most frequently observed consists of two enzymatic processes. In the initial reaction, Trehalose-6-phosphate synthase (TPS) catalyzes the transfer of a glucosyl moiety from UDP-glucose (UDP-Glc) to glucose-6-phosphate (Glc-6-P), resulting in the formation of trehalose-6-phosphate (T6P) and UDP. The enzyme TPP catalyzes the dephosphorylation of T6P, resulting in the production of trehalose during the subsequent step [4].

In the majority of bacterial and eukaryotic organisms, the functions of TPS and TPP are segregated into distinct protein domains. As a result of gene fusion, it has been noted that certain archaea and bacteria, such as *Cytophaga hutchinsonii*, possess proteins that incorporate both active TPS and TPP domains. It is possible to infer that these bifunctional proteins discovered in prokaryotes may have potentially developed into the more complex trehalose biosynthesis enzymes observed in eukaryotes. According to Avonce et al. (2010) [5], there is a hypothesis suggesting that during the course of evolution, it is possible that one or both of these domains in eukaryotic enzymes may have experienced a loss of their catalytic activity.

*Arabidopsis thalian* is recognized for harboring a TPP family of ten smaller proteins, denoted as AtTPPA-J, which consist of 320 to 385 amino acids. The aforementioned sequences are recognized for the presence of the phosphatase box consensus sequences, which are frequently observed in conjunction with the L-2-haloacid dehalogenase (HAD) superfamily of enzymes and include a wide range of phosphatases and hydrolases [4]. Moreover, the AtTPPs exhibit comparable activities, yet they exhibit distinct patterns of differential expression, implying that they may possess a functional association with specific tissues, developmental stages, or biological processes [6].

In fact, certain *AtTPP* genes have recently been associated with the controlling of responses to various environmental stresses. For example, *AtTPPD* is involved in oxidative stress and salt stress resistance [5]. In addition, the two additional isoforms of AtTPP, namely AtTPPF and AtTPPI, have been associated with drought responses [7,8]. On the other hand, the induction of *OsTPP1* and *OsTPP2* in rice (*Oryza sativa*) is observed under cold stress conditions [9,10]. OsTPP7 plays a crucial role in facilitating the plant’s adaptation to anaerobic conditions during germination, a characteristic that has been absent in numerous commercially cultivated varieties [11]. 

Furthermore, a recent study demonstrated that the wheat (*Triticum aestivum* L.) genome harbors a total of 31 *TPP* genes that are dispersed throughout all three genomes and exhibit expression in diverse tissues and in response to distinct stress conditions [12]. Several *TPP* genes in wheat play a role in plant development, specifically in determining the quantity of inflorescence branches and regulating the growth of the root and shoot systems. The upregulation of specific *TPP* genes in wheat has been observed to enhance stress tolerance and enhance growth and yield in the presence of stressful environmental conditions.

The tomato (*Solanum lycrorbreiscum*) genome harbors a total of eight *TPP* genes [13]. The tomato plant possesses duplicated *SlTPP* genes as a result of genome duplication. Nevertheless, it remains uncertain whether all the duplicated genes have preserved their enzymatic functionality. The complete coding sequences of five *SlTPP* genes, namely *SlTPP1*, *SlTPP2*, *SlTPP3*, *SlTPP4*, and *SlTPP8*, were cloned and subsequently assessed for their activity through transformation into yeast. The results of the experiment indicated that all the *SlTPP* genes that were tested exhibited TPP enzymatic activity and were capable of restoring the growth impairment observed in yeast cells that lacked native TPP activity.

In addition, 11 *TPP* genes were identified in quinoa (*Chenopodium quinoa*), and their evolutionary history, conserved domains, and physicochemical characteristics were examined [14]. Analysis of the transcriptome and metabolome revealed that *CqTPP*s contributed to quinoa’s stress response when subjected to saline–alkali conditions.

Pomegranate (*Punica granatum* L.) is a member of the Lythraceae family and is known for its ecological, cultural, and economic significance [15]. Bhantana et al. (2010) highlighted the potential of pomegranate as a valuable candidate for studying perennial deciduous fruit trees [16]. This makes it highly valuable for studying the mechanisms by which plants respond to abiotic stress. The plant exhibits a high degree of tolerance towards diverse soil conditions. The availability of complete genome sequencing data for pomegranate, as well as their publication [17,18,19], has provided essential support for advancing research on the gene function of pomegranate. 

The aim of this study is to utilize bioinformatic tools to identify the members of the TPP family in pomegranate. These members’ gene characteristics, such as length, CDs, chromosomal distribution, and intron–exon structure, will be determined. In addition, PgTPP protein properties such as length, Ip, GRAVY, conserved domains, conserved motifs, synteny, and phylogenetic relationships with Arabidopsis and tomato will be examined. Cis-acting elements in the promoter region and gene expression analysis of *PgTPP* genes under abscisic-acid-mediated drought stress will be evaluated. The findings of this study establish a fundamental basis for the investigation of the cloning and functional analysis of *PgTPP* genes.

## 2. Results

### 2.1. Basic Information of TPP Family Genes in Pomegranate

A total of 22 probable TPP proteins were identified based on HMM profiles. However, using the phosphatase domain (PF03083) to search conserved domain, only 17 proteins were retained. Analysis of the identified proteins showed that a number of proteins are identical (splicing variant of mRNA) and others are isomers. The identified 17 proteins are encoded by only seven genes (Table 1). These genes were named from *PgTPP1* to *PgTPP7*. The amino acid length of the PgTPP family members ranges from 315 aa to 389 aa, with molecular weight (MW) ranging from 35,674 to 43,295 Da. The ranges of genomic DNA (gDNA) and coding sequences (CDS) are 3172–8011 bp and 948~1170 bp, respectively. The subcellular localization prediction revealed that TPP members were present in a wide range of organelles, with three out of seven members specifically located in chloroplasts. Data regarding the IP and GRAVY for each TPP member are all shown in Table 1.

### 2.2. Phylogenetic Tree of Full-Length Pomegranate, Arabidopsis, and Tomato TPP Proteins

**Figure 1:**
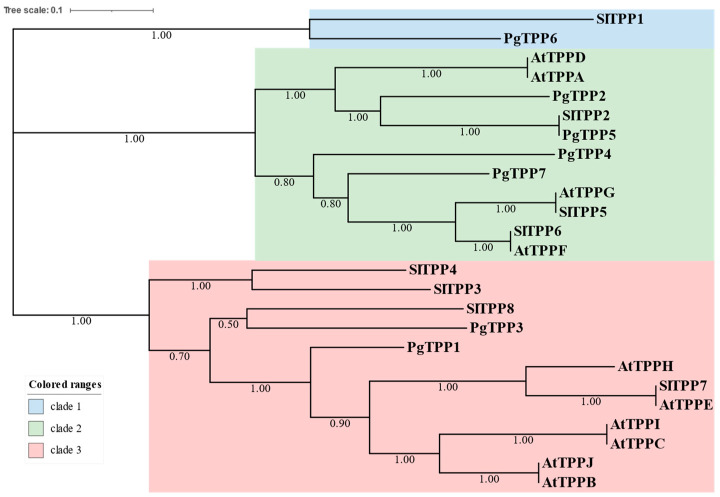
The phylogenetic tree of the TPP family and sequences from *P. granatum*, *A. thaliana*, and *S. lycopersicum*. The clades are presented in various colors. The bootstrap values are indicated.

In order to examine the phylogenetic relationship of the TPPs in pomegranate, the TPP family members of pomegranate were compared to the TPP family members of Arabidopsis and tomato. The findings indicated that members of the TPP family were categorized into three distinct clades, with Clade I consisting of 2 members, Clade II consisting of 11 members, and Clade III consisting of 12 members (Figure 1). Clade I exclusively consisted of PgTPP5 and SlTPP1, whereas Clade II and Clade III included the remaining TPP members from the three species.

### 2.3. Gene Structure and Motif Analysis

**Figure 2:**
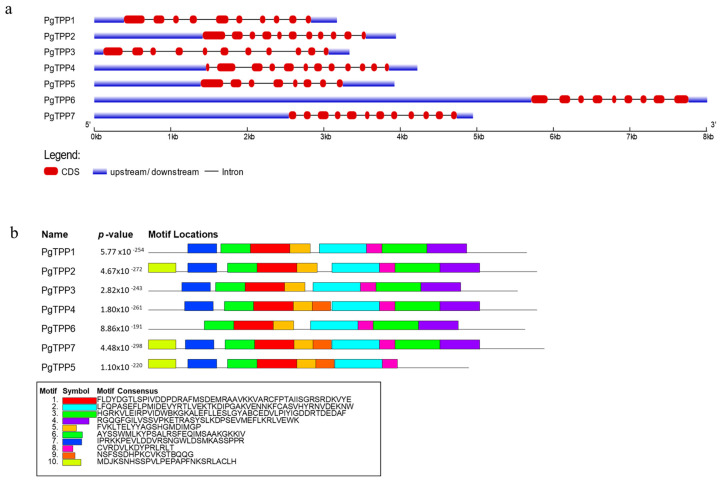
(**a**) The intron/exon structures of the *PgTPP* genes. The dark red boxes represent exons, the black lines represent introns, and the blue boxes represent untranslated regions (UTRs). (**b**) Conserved motifs in the PgTPP proteins. Different colored boxes represented different conserved motifs.

Based on gDNA and CDS sequences, the gene structure of each *PgTPP* gene, including untranslated regions (UTRs), exons, and introns, was predicted. The exon number ranged from 8 to 12 (Figure 2a). The examination of the gene structure revealed that *PgTPP* genes varied significantly in terms of the number of introns. Ten conservative motifs were discovered in PgTPP proteins through the motif distribution analysis. The sequencing data for these conservative motifs, whose lengths varied from 15 to 50 aa, are displayed in Figure 2b. In all PgTPPs, motifs 1, 2, 5, 6, and 8 are present. Only PgTPP5 lacks motifs 3 and 4, while PgTPP6 lacks motif 7. In addition, only PgTPP4, PgTPP5, and PgTPP7 include motif 9, whereas PgTPP2, PgTPP5, and PgTPP7 contain motif 10.

### 2.4. Chromosomal Distribution and Homology Analysis of PgTPP Genes

**Figure 3:**
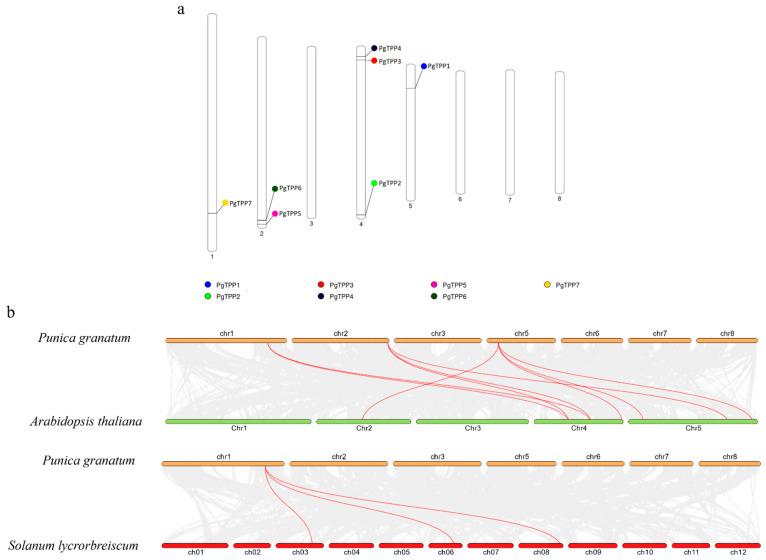
(**a**) *PgTPP* gene distribution on the chromosomes. (**b**) Collinearity analysis of the *TPP* family members in *P. granatum*, *A. thaliana*, and *S. lycpreiscum*. The grey lines in the background illustrate the collinearity of the entire genome, while the red lines highlight the collinearity of the *TPP* genes.

The pomegranate genome has a total of seven *PgTPP* genes, which are distributed over four chromosomes. Chromosome 4 contains three *PgTPP* genes, Chromosome 2 contains two *PgTPP* genes, and Chromosomes 1 and 5 each contain one gene (Figure 3a). A comparative analysis was conducted to investigate the relationship between *TPP* genes in pomegranate, Arabidopsis, and tomato through a synteny analysis. This analysis revealed the presence of nine pairs of syntenic *TPP* genes between pomegranate and Arabidopsis, as well as three pairs of syntenic *TPP* genes between pomegranate and tomato (Figure 3b).

### 2.5. Cis-Acting Regulatory Elements in PgTPP Promoters

**Figure 4:**
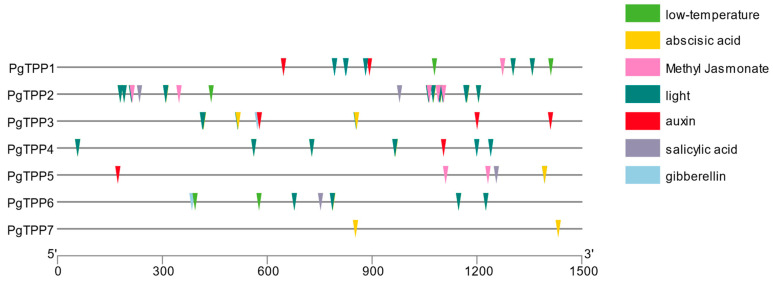
Cis-acting element analysis of the *PgTPP* genes in *P. granatum*. The 1500 bp sequence preceding the coding sequence is shown by the black line, while different colored squares imply distinct cis-acting elements.

Promoters play a vital role in controlling the expression of a gene by mediating the interaction between transcription factors and cis-acting regulatory elements [20]. As a result, evaluating the possible roles of target genes requires a knowledge of how they are controlled, which may be gained by studying upstream regulatory sequences. To thoroughly identify the probable cis-acting regulatory elements, we isolated and searched 1500 bp of non-coding sequences upstream of each *PgTPP* gene. In our study, it was found that the regulatory regions of the *PgTPP* genes exhibited a notable enrichment of hormone-related motifs. Specifically, motifs associated with abscisic acid, auxin, gibberellin, salicylic acid, and methyl jasmonate were observed to be significantly enriched (Figure 4). In addition, light- and low-temperature-responsive elements were detected. 

### 2.6. Expression Profiles of PgTPP Genes under Abscisic-Acid-Mediated Drought Stress

**Figure 5:**
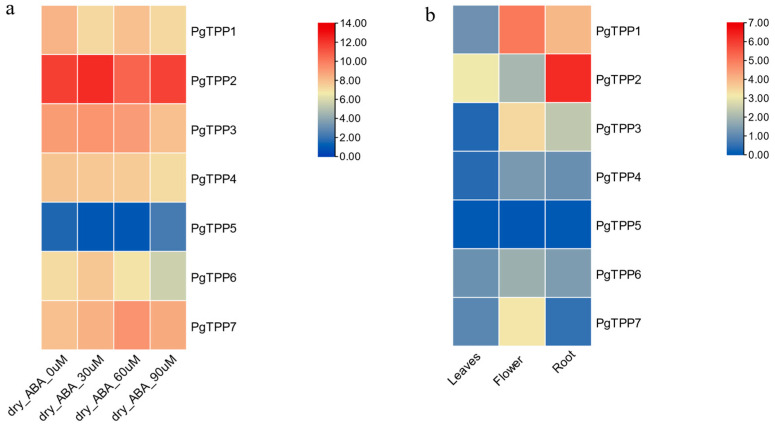
(**a**) Expression profiles of *TPP* genes in *P. granatum* under different abscisic-acid-mediated drought stress. The transcriptomic data were normalized via log2 to generate a heatmap. (**b**) Expression profiles of *TPP* genes in *P. granatum* in root, flower, and leaves. The transcriptomic data were normalized via log2 (TPM + 1) to generate a heatmap.

In this study, publicly accessible RNA-seq data were utilized to examine the expression patterns of *PgTPP* genes in leaves that had undergone abscisic-acid-mediated drought stress (30, 60, and 90 μM) and untreated pomegranates (0 μM as control). This analysis aimed to investigate the potential involvement of *PgTPP* genes in the abscisic-acid-mediated drought stress resistance of pomegranate. There is considerable variation in the levels of expression observed among various *PgTPP* genes when subjected to different treatments. The *PgTPP2* gene exhibits a significantly elevated level. Furthermore, a significant downregulation is observed in the expression of the *PgTPP5* gene. The expression levels of the remaining genes exhibited a moderate degree of activity (Figure 5a).

### 2.7. Tissue-Differential Gene Expression Patterns of PgTPPs

The analysis of the tissue-specific gene expression patterns of *PgTPP*s revealed that in the root tissue, only *PgTPP2* exhibited significantly higher expression levels. *PgTPP1* displayed moderate expression, whereas the remaining *PgTPP*s (*PgTPP3*, *PgTPP4*, *PgTPP5*, *PgTPP6*, and *PgTPP7*) were downregulated (Figure 5b). Within the floral tissue, no expression of *PgTPP3* and *PgTPP7* was found, while *PgTPP1* exhibited a moderate level of expression. Conversely, the remaining members of the *TPP* gene family displayed a downregulated expression pattern. In the tissue of leaves, it was observed that all seven *PgTPPs* exhibited downregulation, with the exception of *PgTPP2*, which displayed no detectable expression.

## 3. Discussion

T6P is a signal metabolite that has been shown to play a crucial role in plant metabolism, growth, and development. Thus, further research into the mechanisms and functions of various plant TPP proteins is urgently required. The plant-specific trehalose-6-phosphate phosphatases (TPPs) are integral to the biosynthesis of trehalose and hold significant importance in both plant development and the response to stress. Hence, the trehalose biosynthetic pathway and its corresponding gene products exhibit potential as viable targets for enhancing plant performance in the presence of stressful conditions. In the current investigation, a total of seven *TPP* genes were identified in the pomegranate species.

Certain characteristics of *PgTPP* genes were evaluated in this investigation. Like the TPPs found in other plant species, the proteins encoded by the vast majority of *PgTPP* genes have a projected molecular mass of 35,674–42,850 Da and an IP of around 6.0–9.3. The length of the PgTPP proteins varied between 315 and 389. This finding aligns with previous research, such as the study conducted by Du et al. (2022) [12], which reported a length of approximately 350 for TPP proteins in wheat, and the study by Wang et al. (2023) [14], which observed a range of 324 to 387 for TPP proteins in quinoa.

With the release of full-genome sequencing, genetic study of the *TPP* gene family has revealed the presence of 10 *TPP* genes in Arabidopsis [21], 11 in maize [22], 13 in rice [23], 30 in wheat [12], 8 in tomato [13], 11 in quinoa [14] and 79 in cotton [14]. There was significant variation observed in the number of *TPP* genes across a variety of plants.

In addition, the observed diversity in protein sequences of *TPP* genes suggests that there might be a regulatory function for TPP proteins. This implies that plants may have the ability to modulate T6P levels in response to specific requirements or environmental conditions. In addition, the analysis of subcellular localization indicated that PgTPP members were distributed across various organelles, with a subset of three out of seven members being specifically localized within chloroplasts. This finding indicates the diverse and complex functions of TPP proteins.

The PgTPP protein family was categorized into three distinct subclades through a comparison of their relationship with homologous TPPs found in other species, such as Arabidopsis and tomato. Indeed, several *AtTPP* genes were correlated with an abiotic stress response. For instance, it has been reported that *AtTPPD* plays a crucial role in conferring resistance to salt and oxidative stress [6]. Additionally, *AtTPPF* and *AtTPPI* have been implicated in the response to drought conditions [6,9]. Additionally, it has been found that the rice genes *OsTPP1* and *OsTPP2* play a role in the response to cold stress [10,11], while *OsTPP7* is responsible for regulating resistance to anaerobiosis during the process of germination [11]. The level of expression of *CqTPP4* in quinoa leaves exhibited an apparent reduction in response to saline–alkali stress conditions [14]. 

If plants encounter stress, specific transcription factors (TFs) are triggered to bind to certain cis-acting elements. The binding process described herein serves to modulate the transcriptional activity of stress response genes located downstream. The present investigation has successfully identified a number of cis-acting elements that are linked to stress response, phytohormone signaling, and light regulation within the promoter regions of *PgTPP* genes. These cis-acting elements include abscisic-acid-, auxin-, gibberellin-, salicylic-acid-, methyl-jasmonate-, light- and low-temperature-responsive elements. 

The expression levels of *PgTPP2* and *PgTPP5* were significantly altered in pomegranate leaves subjected to abscisic-acid-mediated drought stress at concentrations of 30, 60, and 90 μM, compared to untreated pomegranates (0 μM as control). The gene *PgTPP2* exhibited upregulation and is distinguished from other *PgTPP* genes by the absence of the ABA element in its promoter region. This observation implies a potential association between *PgTPP2* and the response to drought stress. The remaining five *PgTPP* genes exhibited no statistically significant response when subjected to drought stress mediated by abscisic acid. In addition, the existence of an abscisic acid cis-acting element on the remaining *PgTPP* genes in plants could potentially play a role in suppressing gene expression in response to drought mediated by ABA. Hence, certain *TPP* genes exhibit upregulation while others undergo downregulation. The observed phenomenon may be attributed to the fact that certain *TPP* genes play a crucial role in regulating plant growth and development, while others are associated with the plant’s response to stress.

The investigation of *TPP* family genes in pomegranate is crucial for advancing research on how pomegranate responds to environmental stress, its growth and development, and enhancing its yield in challenging conditions. Enhancing plant stress resistance is a complicated biological process influenced by multiple genes. Consequently, the impact of introducing a single gene to enhance plant stress resistance is constrained. Additionally, the incorporation and utilization of *TPP* gene promoters may potentially address the issue of precise alterations in *TPP* gene expression. Additionally, the utilization of trehalose defense response in breeding high-performing varieties under conditions of drought and salt stress has emerged as a prominent area of research interest. As our understanding of the specific biological function of trehalose continues to grow, it is anticipated that trehalose will increasingly contribute to enhancing resistance in crops through breeding efforts.

## 4. Materials and Methods

### 4.1. Genome-Wide Identification and Annotation of TPP Genes in Pomegranate 

The protein sequences of the *TPP* genes in Arabidopsis were obtained from the Arabidopsis Information Resource (TAIR) database (http://www.arabidopsis.org/ accessed on 1 June 2023) [24]. These proteins were aligned, and the output was used to build a Hidden Markov Model (HMM), which was used to search for homologous proteins in pomegranate using HMMER 3.3.2 [25]. The genomic assembly of pomegranate was downloaded from NCBI (ASM765513v2). The putative TPP conserved protein domains were verified by utilizing the Conserved Domain Database (CDD) (https://www.ncbi.nlm.nih.gov/cdd accessed on 10 June 2023) [26]. Data regarding the isoelectric point (IP), grand average of hydropathicity (GRAVY), and relative molecular mass of the PgTPP proteins were acquired through the website https://web.expasy.org/compute_pi/ (accessed on 10 June 2023). The subcellular localization of PgTPP proteins was predicted using the Plant-mSubP online tool https://bioinfo.usu.edu/Plant-mSubP/ (accessed on 10 June 2023) [27].

### 4.2. Phylogenetic Tree of Full-Length Pomegranate, Arabidopsis, and Tomato

TPP proteins were generated with raxmlGUI 2.0 and the maximum likelihood method with 1000 bootstrap values. To build the phylogenetic tree, the protein sequences of *TPP* genes from *pomegranate*, *Arabidopsis*, and *tomato* were used. MAFFT v7.402 was used to align all TPP sequences [28]. The phylogenetic analyses were performed using raxmlGUI with 1000 bootstrap repetitions and GTR + G, maximum likelihood [29]. 

### 4.3. Gene Structure and Conserved Motif Analysis

Gene structure data for *PgTPP* genes were obtained from the (ASM765513v2) reference genome and processed using the Gene Structure Display Server 2.0 (GSDS; http://gsds.cbi.pku.edu.cn/ accessed on 12 June 2023). The proteins of *PgTPP* gene family were examined for conserved motifs through the utilization of the MEME online tool (http://meme-suite.org/ accessed on 12 June 2023) [30]. 

### 4.4. Chromosomal Distribution and Homology Analysis of TPP Genes

Chromosomal distribution maps were generated using the PhenoGram online tool (http://visualization.ritchielab.org/phenograms/plot accessed on 12 June 2023) based on the information of the *PgTPP* family genes [31]. The collinear genes between pomegranate and Arabidopsis and tomato were extracted using the TBtools program, and then they were mapped using a dual synteny plot [32].

### 4.5. Prediction and Analysis of Cis-Acting Elements in Promoter Regions

The 1500 bp sequences upstream of all *TPP* genes were extracted as candidate promoter sequences and used for cis-acting elements, which was achieved by utilizing the PlantCare web tool (https://bioinformatics.psb.ugent.be/webtools/plantcare/html/ accessed on 13 June 2023) [20]. 

### 4.6. Expression Profile Analysis of the TPP Gene Family in Different Tissues and in Leaves of Pomegranate under Abscisic-Acid-Mediated Drought Stress

In order to examine the expression patterns of *PgTPP* genes in pomegranate leaves under abscisic-acid-mediated drought stress, transcriptome data were obtained from the NCBI GEO database and the expression patterns of *PgTPP* across various tissues and organs were employed (http://www.ncbi.nlm.nih.gov/ accessed on 15 June 2023).

In this experiment, following a growth period of 90 days, seedlings that exhibited healthy development under identical growth conditions were relocated to an artificial incubator. The incubator maintained a temperature range of 26–32 °C and provided natural light conditions. The plants were exposed to drought stress through the alteration of soil moisture content in the cultivation pot, which was reduced to approximately 30–35% of the field water capacity. The weighing technique was employed to regulate soil moisture levels in containers. Simultaneously with the occurrence of drought stress, the plants were divided into four distinct experimental groups. Among these groups, three were subjected to daily spraying with solutions containing concentrations of 30, 60, and 90 μM ABA, respectively, while the remaining group served as the control and was treated with an equivalent volume of distilled water (0 μM ABA solution).

Transcript data from three distinct tissue samples of pomegranate were successfully acquired (SRR5279396, SRR5279395, SRR5279397). FASTQC was used to filter the RNA-Seq for quality [33]. A Trimmomatic tool was used for trimming adaptors from the dataset [34]. The RNA-seq were then aligned to the pomegranate reference genome (ASM765513v2) using HISAT2 (Version 2.2.1) [35]. For quantifying abundances of transcripts from RNA-Seq data, kallisto (Version 0.48.0) was used [35]. Tbtools was used to illustrate the values of log2 (TPM + 1) via a heatmap [32].

## 5. Conclusions

In this research, a total of seven putative *PgTPP* genes have been successfully identified within the genome of pomegranate. Physicochemical characteristics, gene structures, conservative domains and motifs in proteins, and cis-regulatory regions were comprehensively examined. The evolutionary relationships between these genes and *TPP* genes in Arabidopsis and tomato have also been investigated.

The findings indicate that there is a notable degree of conservation within the TPP family present in pomegranate. The findings from the transcriptome analyses demonstrate that the *PgTPP2* and *PgTPP5* genes in the leaves of pomegranate are involved in the response to drought stress, highlighting their notable contribution in this biological process. The present study possesses the capacity to elucidate the biological capabilities of PgTPP proteins in diverse developmental phenomena and responses to stresses in the pomegranate plant. Nevertheless, there remains a need for additional investigation pertaining to their exact biological function. Therefore, a full functional validation investigation of PgTPPs might help produce agricultural cultivars that are resistant to stress.

## Figures and Tables

**Table 1:** The identification and sequence analysis of *TPP* gene family in pomegranate.

Locus_ID	Gene Name	Protein _ID	Transcript_ID	Location, Start–End	Strand	Gene Length	CDS (bp)	Protein Length (A.A)	Protein Molecular Weight (Da)	PI	GRAVY	No of Exons	Cellular Localization Prediction
LOC116206686	*PgTPP1*	XP_031395344.1	XM_031539484.1	Chromosome 5, NC_045131.1 (5264138..5267309)	−	3172	1119	372	41,950	9.46	−0.405	11	Plastid
LOC116206238	*PgTPP2*	XP_031394912.1	XM_031539052.1	Chromosome 4 NC_045130.1 (39635185..39639127)	−	3943	1149	382	42,707	9.04	−0.283	12	Plastid
LOC116206238	*PgTPP2*	XP_031394913.1	XM_031539053.1	Chromosome 4 NC_045130.1 (39635185..39639127)	−	3943	1149	382	42,707	9.04	−0.283	12	Plastid
LOC116202804	*PgTPP3*	XP_031390275.1	XM_031534415.1	Chromosome 4 NC_045130.1 (2818814..2822149)	+	3336	1092	363	40,638	9.3	−0.291	11	Nuclei
LOC116202804	*PgTPP3*	XP_031390276.1	XM_031534416.1	Chromosome 4 NC_045130.1 (2818814..2822149)	+	3336	1083	360	40,283	9.24	−0.304	11	Nuclei
LOC116202804	*PgTPP3*	XP_031390277.1	XM_031534417.1	Chromosome 4 NC_045130.1 (2818814..2822149)	+	3336	1035	344	38,591	9.44	−0.313	10	Nuclei
LOC116202714	*PgTPP4*	XP_031390179.1	XM_031534319.1	Chromosome 4 NC_045130.1 (2007596..2011818)	+	4223	1149	382	42,190	6.17	−0.318	14	Mitochondria
LOC116202714	*PgTPP4*	XP_031390180.1	XM_031534322.1	Chromosome 4 NC_045130.1 (2007596..2011818)	+	4223	1149	382	42,850	6.03	−0.335	14	Mitochondria
LOC116202714	*PgTPP4*	XP_031390181.1	XM_031534320.1	Chromosome 4 NC_045130.1 (2007596..2011818)	+	4223	1149	382	42,850	6.03	−0.335	14	Mitochondria
LOC116202714	*PgTPP4*	XP_031390182.1	XM_031534321.1	Chromosome 4 NC_045130.1 (2007596..2011818)	+	4223	1149	382	42,850	6.03	−0.335	13	Mitochondria
LOC116202714	*PgTPP4*	XP_031390184.1	XM_031534324.1	Chromosome 4 NC_045130.1 (2007596..2011818)	+	4223	1131	376	42,850	6.03	−0.335	14	Mitochondria
LOC116194837	*PgTPP5*	XP_031379593.1	XM_031523735.1	Chromosome 2 NC_045128.1 (44074459..44078382)	−	3924	948	315	35,674	9.31	−0.37	13	Plastid
LOC116194837	*PgTPP5*	XP_031379594.1	XM_031523734.1	Chromosome 2 NC_045128.1 (44074459..44078382 complement)	−	3924	948	315	35,674	9.31	−0.37	12	Plastid
LOC116194837	*PgTPP5*	XP_031379595.1	XM_031523733.1	Chromosome 2 NC_045128.1 (44074459..44078382)	−	3924	948	315	35,674	9.31	−0.37	12	Plastid
LOC116194202	*PgTPP6*	XP_031378813.1	XM_031522953.1	Chromosome 2 NC_045128.1 (43096590..43104600)	+	8011	1113	370	41,565	9.16	−0.4	10	Golgi
LOC116193147	*PgTPP7*	XP_031377785.1	XM_031521925.1	Chromosome 1 NC_045127.1 (46957219..46962168)	+	4950	1071	356	39,602	6.8	−0.303	14	Nuclei
LOC116193147	*PgTPP7*	XP_031377784.1	XM_031521924.1	Chromosome 1 NC_045127.1 (46957219..46962168)	+	4950	1170	389	43,295	8.58	−0.358	13	Nuclei

## Data Availability

All the data created or analyzed for this investigation are presented in this publication.
